# HLA class, calcineurin inhibitor levels, and the risk of graft failure in kidney recipients with *de novo* donor-specific antibodies

**DOI:** 10.3389/fimmu.2024.1493878

**Published:** 2024-11-20

**Authors:** Marc-Antoine Béland, Isabelle Lapointe, Isabelle Côté, Julie Lesage, Isabelle Houde, Eric Wagner, Julie Riopel, Eva Latulippe, Olivier Désy, Sacha A. De Serres

**Affiliations:** ^1^ Transplantation Unit, Renal Division, Department of Medicine, University Health Center (CHU) of Quebec, Faculty of Medicine, Laval University, Quebec, QC, Canada; ^2^ Immunology and Histocompatibility Laboratory, University Health Center (CHU) of Quebec, Faculty of Medicine, Laval University, Quebec, QC, Canada; ^3^ Department of Pathology, University Health Center (CHU) of Quebec, Faculty of Medicine, Laval University, Quebec, QC, Canada

**Keywords:** donor-specific alloantibodies, kidney transplantation, anti-HLA alloantibodies, graft loss, HLA class 1

## Abstract

**Introduction:**

De novo donor-specific HLA antibody (dnDSA) are associated with poor outcomes. Whether this observation applies to both HLA class I and II dnDSA remains unclear.

**Methods:**

We studied 1236 consecutive kidney recipients who had routine anti-HLA antibody surveillance post-transplant.

**Results:**

During the screening period, 55/1236 (4.4%) patients developed dnDSA: 18 (33%) HLA-I only, 33 (60%) HLA-II only, and 4 (7%) both classes. Thirty patients experienced graft loss at a median of 39 months after dnDSA detection: 9/18 (50%) HLA-I only, 17/33 (52%) HLA-II only, and 4/4 (100%) both classes. A control group was created by matching patients with dnDSA to patients who did not develop DSA and had a functioning graft at the time of dnDSA detection in their respective cases. Compared with these controls, the risk estimates of graft loss were similar between patients with HLA-I only and HLA-II only dnDSA (aHR [95% CI] 2.7 [1.1-6.6], p=0.04 and 3.1 [1.5-6.6], p<0.01 respectively). Additionally, the risk of graft loss decreased with increasing CNI trough levels following dnDSA detection (aHR 0.7 [0.6-0.9] for each increase in 1 ng/mL, p=0.02).

**Conclusions:**

The prognosis of patients with dnDSA is similar regardless of the HLA class specificity. Lower calcineurin inhibitor levels predict graft loss in such patients.

## Introduction

The development of *de novo* donor-specific HLA antibodies (dnDSAs) is associated with poorer outcomes in kidney transplant recipients (1, 2). The decline in estimated glomerular filtration rate (eGFR) accelerates post-dnDSA development, leading to a decrease in post-transplant graft survival (3). There is an increasing consensus in the renal transplant community that HLA class II dnDSAs are associated with negative and worse outcomes than HLA class I dnDSAs. This notion is mostly extrapolated from data published in the early 2000s, showing that HLA class II DSAs are more frequently associated with antibody-mediated rejection (AMR) and transplant glomerulopathy than HLA class I DSAs (2, 4, 5).

Despite its association with poor prognosis, a clinical recommendation article published last year suggested that the utility of post-transplant dnDSA detection remains unclear (6). The development of dnDSAs does not necessarily lead to graft rejection (2, 7–9). Moreover, clear evidence to support any therapy for AMR is currently lacking (10). To define how dnDSA detection could impact maintenance immunosuppression, we previously examined the association between calcineurin inhibitor (CNI) levels following dnDSA detection and outcomes. We observed a positive association between higher CNI levels and better graft survival in patients with dnDSA (8). Similarly, high intra-patient variability in tacrolimus trough levels was associated with inferior graft survival following the diagnosis of chronic AMR (11). In stable patients, tacrolimus levels below 5 ng/mL were a predictor of dnDSA development (12). Collectively, the data suggested that longitudinal dnDSA detection might be relevant to optimize CNI levels in patients with otherwise stable graft function.

This study primarily aimed to assess the association between HLA class I versus class II dnDSA specificity and graft outcomes. The secondary aim was to determine whether the association between CNI trough levels and graft outcomes following dnDSA detection could be confirmed.

## Patients and methods

### Study design and population

This was a single-center, observational, retrospective cohort study. Circulating anti-HLA alloantibodies were prospectively detected in 1236 consecutive patients who underwent kidney transplantation between January 2000 and December 2021. The DSA screening period spanned from January 2005 to December 2022. Anti-HLA antibody screening was routinely performed at 0, 1, 3, 6, and 12 months post-transplant and then annually as part of a routine surveillance protocol. Anti-HLA antibody detection was also conducted at the time of biopsy or following any sensitizing events. None of the patients had DSA at the time of transplantation. Patients in whom dnDSA was detected after graft loss were excluded from the study.

In addition to examining the cohort with dnDSAs, a matched control group was constructed comprising patients without dnDSAs. Controls were randomly sampled at a 1:1 ratio from the source population based on the following matching criteria: age (± 5 years), rank of transplant, and date of transplantation (± 6 months). To ensure that controls were sampled from an at-risk population, patients were considered potential controls only if they had a functioning graft at the time of dnDSA detection in the corresponding case.

### Anti-HLA antibody assessment

Serum samples were screened for dnDSA by flow cytometry using FlowPRA beads (One Lambda, Canoga Park, CA, USA). Between 2005 and 2012, the identification was performed using single-flow antigen beads. Since 2012, the Luminex Platform has been used to identify HLA antibodies using LABScreen single-antigen beads (One Lambda). A normalized mean fluorescence intensity (MFI) cut-off value of ≥ 1500 was used as a guide to detect dnDSA and their presence was confirmed in each case by the HLA laboratory director by eplet analysis. When donor HLA-DQB1 typing data were unavailable, HLA-DQB1 typing was assigned based on frequency associations within the donor ethnic group.

### Measurement of CNI exposure

Daily average tacrolimus exposure was calculated based on the blood tacrolimus levels measured at 0, 1, 3, 6, 12, and 24 months after dnDSA detection. Four patients received cyclosporine rather than tacrolimus. In such cases, C2 blood levels were converted to C0 tacrolimus equivalents using a 1/115 correction factor (8, 13, 14). Four patients did not receive tacrolimus at the time of DSA detection but were administered medication afterward.

### Pathologic classification

Biopsy samples were prospectively graded by local attending renal pathologists (E.L. and J.R.) according to the Banff 1997 criteria and their subsequent updates; the diagnosis of borderline (BL) changes suspicious for acute T cell-mediated rejection (TCMR) was made using the Banff 2019 definition (15–19). All biopsies were reviewed by pathologists and nephrologists during weekly clinicopathological transplant conferences in which challenging cases were discussed.

### Study approval

This study was approved by our Institutional Ethics Committee (Project 2024-6949). The reported clinical and research activities are consistent with the Principles of the Declaration of Istanbul.

### Statistical analysis

The Mann–Whitney test, Fisher’s exact test, or Chi-squared test was used to compare baseline clinical characteristics between patients according to the HLA class of dnDSAs. The relationship between HLA class of dnDSA, tacrolimus levels and endpoints was assessed using the Kaplan–Meier method, log-rank test, and Cox proportional hazard model. When patients had more than one DSA, the date of appearance of the first dnDSA was used as a reference to analyze time post-dnDSA. Statistical analyses were performed using STATA version 11.0 (StataCorp, College Station, TX, USA) and SPSS Statistics version 29.0 (IBM Corp., Armonk, NY, USA). All tests were two-tailed, and a p-value <0.05 was considered statistically significant.

## Results

### Study population

dnDSA was detected in the sera of 55 patients during the screening period. These patients underwent kidney transplantation between January 2000 and December 2021, during which a total of 1236 patients received a kidney. The incidence of dnDSAs in the entire cohort was thus 4.4% during the period, with an incidence ratio of 0.46/100 person-years. The patients were predominantly recipients of first kidney transplant from a deceased donor (**Table 1**).

**Table 1:** Clinical and pathological characteristics of the population according to HLA class of dnDSA.

	All dnDSA positive (n=55)	HLA class I only (n=18)	HLA class II only (n=33)	HLA classes I and II (n=4)	p-value
Age (yr)	49 ± 15	54 ± 11	46 ± 17	48 ± 15	0.22
Female gender	23 (42)	8 (44)	12 (36)	3 (75)	0.32
First transplant	49 (89)	17 (94)	29 (88)	3 (75)	0.50
Deceased donor	48 (87)	18 (100)	27 (82)	3 (75)	0.13
Delayed graft function	13 (25)	7 (39)	5 (16)	1 (25)	0.20
Serum creatinine (μmol/L)	111 [93, 178]	116 [92, 200]	110 [93, 168]	113 [95, 165]	0.91
Time post-transplant (mo)	61 [28, 101]	48 [11, 96]	80 [38, 126]	30 [15, 36]	0.07
HLA mismatch					
A mismatch	1.2 ± 0.8	1.3 ± 0.7	1.1 ± 0.8	1.5 ± 0.6	0.32
B mismatch	1.2 ± 0.7	1.4 ± 0.6	1.1 ± 0.7	1.3 ± 0.5	0.15
DRB1 mismatch	1.0 ± 0.7	0.7 ± 0.8	1.1 ± 0.6	1.0 ± 0.8	0.19
DQB1 mismatch	0.9 ± 0.7	0.5 ± 0.5	1.2 ± 0.7	1.0 ± 0.0	0.05
cPRA^a^	10 ± 26	17 ± 30	5 ± 20	19 ± 34	0.24
0-5%	42 (81)	13 (72)	26 (87)	3 (75)	
6-85%	9 (17)	5 (28)	3 (10)	1 (25)	0.52^b^
>85%	1 (2)	0 (0)	1 (3)	0 (0)	
Induction					0.37
ATG	6 (11)	3 (18)	2 (6)	1 (25)	
IL2RI	40 (73)	14 (78)	24 (73)	2 (50)	
Prednisone dose at detection, mg	6.8 ± 2.5	6.5 ± 1.9	7.2 ± 2.8	5.0 ± 0	0.19
TAC level at detection, ng/ml	5.5 ± 2.5	5.6 ± 2.3	5.3 ± 2.6	5.9 ± 3.7	0.89
Mycophenolate dose at detection, mg^c^	1057 ± 614	1152 ± 635	1005 ± 599	1063 ± 774	0.72
Banff scores					
	(n=34)	(n=11)	(n=19)	(n=4)	
t	1.2 ± 0.8	1.3 ± 0.8	1.1 ± 0.8	1.4 ± 1.1	0.71
i	0.8 ± 1.0	0.8 ± 0.9	0.7 ± 0.9	1.5 ± 1.5	0.30
v	0.1 ± 0.3	<0.1 ± 0.1	0.2 ± 0.4	0.0 ± 0.0	0.47
g	1.0 ± 1.0	1.0 ± 1.3	1.2 ± 1.0	0.5 ± 0.6	0.45
ptc	0.7 ± 1.0	0.7 ± 1.2	0.7 ± 1.1	0.9 ± 1.2	0.96
g + ptc	1.6 ± 1.6	1.7 ± 2.1	1.9 ± 1.4	1.4 ± 1.7	0.82
C4d IH	0.4 ± 0.8	0.3 ± 0.6	0.4 ± 0.9	0.3 ± 0.5	0.85
C4d IF	0.5 ± 0.9	0.4 ± 0.8	0.5 ± 0.9	1.0 ± 1.4	0.55
cg	0.8 ± 1.2	1.0 ± 1.2	0.7 ± 1.3	0.8 ± 1.5	0.86
ct	1.6 ± 0.7	1.8 ± 0.6	1.3 ± 0.6	1.9 ± 1.0	0.10
ci	1.6 ± 0.8	2.0 ± 0.4	1.3 ± 0.8	1.5 ± 1.0	0.07
cv	1.7 ± 0.8	1.8 ± 0.6	1.6 ± 1.0	2.1 ± 0.6	0.55
ah	2.1 ± 0.8	2.2 ± 0.8	2.1 ± 0.8	2.0 ± 1.2	0.91
Rejection^d^					0.61
No rejection	10 (29)	4 (36)	4 (21)	2 (50)	
AMR only	3 (9)	0 (0)	3 (16)	0 (0)	
TCMR/BL only	6 (18)	2 (18)	4 (21)	0 (0)	
AMR + TCMR/BL	15 (44)	5 (45)	8 (42)	2 (50)	

Data are provided as mean ± SD, n (%) or median [25th, 75th percentiles]. Comparisons were performed using ANOVA or Chi square test. Biopsy scores are provided according to the Banff classification (0-3): t, tubulitis; i, interstitial infiltration; v, intimal arteritis; g, glomerulitis; ptc, peritubular capillarities; cg, transplant glomerulopathy; ct, tubular atrophy; ci, interstitial fibrosis; cv, fibrous intimal thickening; ah, arteriolar hyaline thickening; C4d IH, deposition of the C4d fragment of complement component C4 examined by immunohistochemistry; C4d IF, C4d examined by immunofluorescence; IL2RI, Il-2 receptor inhibitor; AMR, antibody-mediated rejection; TCMR, T cell-mediated rejection; BL, borderline changes suspicious for TCMR.

a FlowPRA/Luminex was available in 43 patients, PRA was available by complement-dependent cytotoxicity assay CDC in 9 patients and data was unavailable in 3 patients.

b P-value for the chi-square test for the 3 groups.

c In mycophenolate mofetil equivalent; 4 patients were on azathioprine; 8 patients did not receive antimetabolite, they were included in the calculation of the mean dose.

d 34 patients were biopsied; the percentages for this section are a proportion of the patients biopsied.

### dnDSA to HLA class I versus HLA class II

The median time to dnDSA detection was 61 months post-transplant (25^th^–75^th^ percentiles, 28–101). In total, 18 (33%) patients had HLA class I dnDSAs only, 33 (60%) had HLA class II dnDSAs only, and 4 (7%) had dnDSAs of both HLA classes (**Table 1**). There were no differences in baseline clinical characteristics between these groups of patients, except for a non-significant but clear trend towards shorter time to dnDSA detection post-transplant in patients with both HLA class I and II dnDSAs.

Among the 34 patients who underwent biopsy at the time of dnDSA detection, 20 biopsies were done solely because of the DSA. There was no difference in pathological scores between those with dnDSAs of HLA class I only versus HLA class II only versus both HLA classes (**Table 1**). Overall, rejection was observed in 24/34 (71%) of the patients who underwent biopsy: 3/34 (9%) with AMR only, 6/34 (18%) with TCMR/BL only, and 15/34 (44%) with mixed rejection. We observed no difference in the proportion of patients with a diagnosis of rejection between the HLA class specificities of dnDSA, whether class I, class II or classes I and II.

### Outcomes of patients with dnDSA HLA class I versus class II

**Figure 1:**
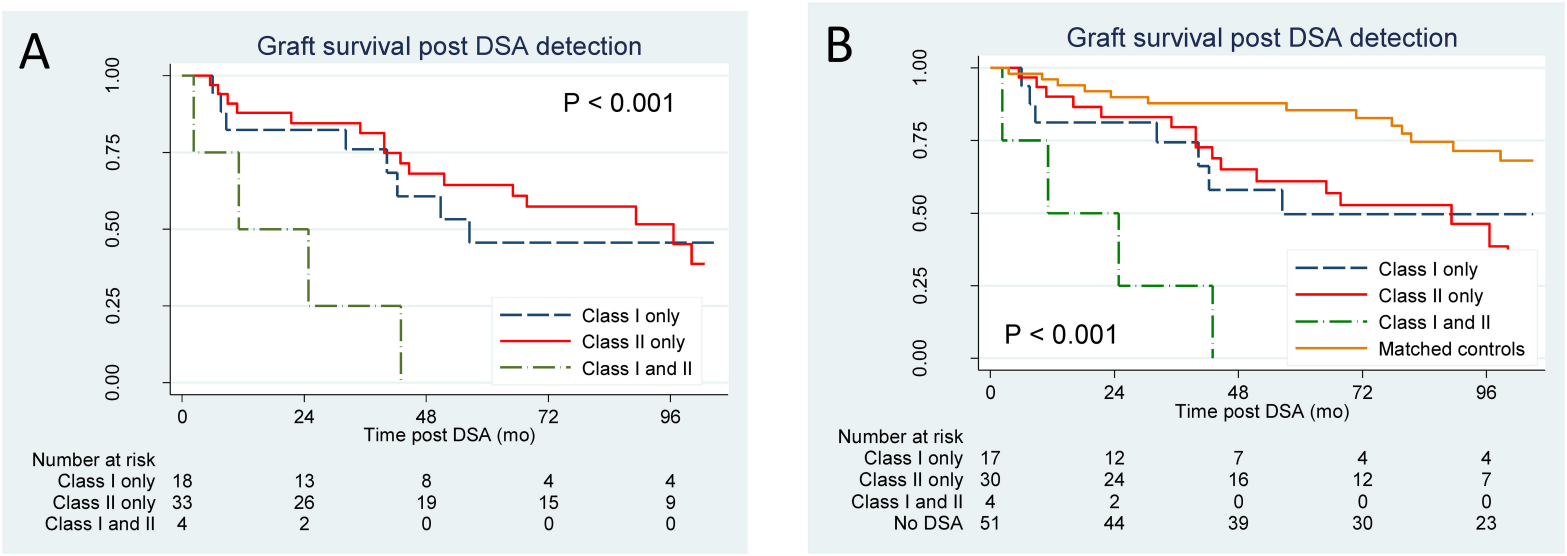
Kaplan–Meier plots for graft loss according to the dnDSA status. **(A)** Analysis of all patients with dnDSA (n=55). **(B)** Analysis of patients following successful matching (total n=102; n=17 HLA class I only, n=30 HLA class II only, n=4 HLA both classes, n=51 controls). Curves were compared using the log-rank test.

During follow-up after dnDSA detection, 30 patients experienced graft loss: 9/18 (50%) with HLA class I dnDSAs only, 17/33 (52%) with HLA class II dnDSAs only, and 4/4 (100%) with dnDSAs of both HLA classes. The median follow-up time was 39 months in patients who experienced graft loss [25^th^–75^th^ percentiles, 10–59 months] versus 79 months in those who retained a functioning graft [39–105 months]. Survival analysis using Kaplan–Meier curves and log-rank tests showed that, compared with the presence of dnDSAs of HLA class I only or HLA class II only, the presence of DSAs of both HLA classes I and II substantially increased the risk of graft loss (**Figure 1A**). In contrast, survival was comparable for those with DSAs of either class I or class II only. Cox modeling suggested that the presence of dnDSA of both HLA classes was a strong predictor of graft loss, even after adjustment for age, sex, serum creatinine level at DSA detection, and time post-transplant at DSA detection (adjusted hazard ratio [aHR] 10.3, 95% CI 2.5–42.2, p<0.01; **Table 2**). These data did not indicate a different prognosis for patients with dnDSAs of HLA class I only compared with those with dnDSAs of HLA class II only (aHR 1.2, 95% CI 0.5–3.0, p=0.68; **Table 2**).

**Table 2:** Univariate and multivariate risk estimates for graft loss associated with each HLA class of dnDSA.

	unadjusted		adjusted^a^	
	Hazard ratio (95% CI)	p-value	Hazard ratio (95% CI)	p-value
Class I only	ref	–	ref	–
Class II only	0.8 (0.4-1.8)	0.60	1.2 (0.5-3.0)	0.68
Classes I and II	4.8 (1.4-16.4)	0.01	10.3 (2.5-42.2)	<0.01

a adjusted for age, sex, serum creatinine at dnDSA detection, time post-transplant at dnDSA detection. n = 55.

Twenty-eight patients had a biopsy during their follow-up after dnDSA detection. Among them, 9 patients had evidence of *de novo* AMR; 6 of those patients experienced graft loss during follow-up. In addition, 13 patients had signs of AMR at the time of dnDSA detection, that persisted on the follow-up biopsy; 7 of them lost their graft. 6 patients had no signs of rejection on the biopsy performed after dnDSA detection.

### Outcomes of patients with dnDSA versus matched controls

To further assess the prognosis of patients with dnDSA, we established a control group without dnDSA. Using the criteria mentioned previously, 51 patients were successfully matched. No major differences were observed in the baseline characteristics between cases and controls (**Supplementary Table S1**). Kaplan–Meier curves and log-rank analysis revealed that compared with matched controls, patients with dnDSA had substantially shorter graft survival (**Figure 1B**). Similarly, adjusted models showed that all groups with dnDSAs had higher risk estimates for graft loss than the matched controls (**Table 3**). Additionally, compared with the controls, the risk estimates for graft loss were similar between patients with dnDSAs of HLA class I only and those with dnDSA of HLA class II only (aHR 2.7, 95% CI 1.1–6.6; p=0.04, and aHR, 3.1, 95% CI, 1.5–6.6; p<0.01 respectively; **Table 3**).

**Table 3:** Univariate and multivariate risk estimates for graft loss associated with each HLA class of dnDSA compared to matched controls.

	unadjusted		adjusted^a^	
	Hazard ratio (95% CI)	p-value	Hazard ratio (95% CI)	p-value
No dnDSA	ref	–	ref	–
Class I only	2.8 (1.2-6.7)	0.02	2.7 (1.1-6.6)	0.04
Class II only	2.6 (1.3-5.2)	<0.01	3.1 (1.5-6.6)	<0.01
Classes I and II	14.1 (4.4-45.2)	<0.01	26.5 (7.1-98.8)	<0.01

a adjusted for age, sex, serum creatinine at dnDSA detection, time post transplant at dnDSA detection. n = 102.

### Association between tacrolimus levels and graft loss

Next, we examined whether the previous findings regarding the association between tacrolimus trough levels and outcomes in patients with dnDSA were valid in the current cohort (n=55). At the time of dnDSA detection, the mean (± standard deviation) tacrolimus level was 5.4±2.5 ng/ml for the entire cohort. For each patient, the average daily exposure to tacrolimus was derived from the blood levels at time 0, 3, 6, 12, and 24 months after DSA detection. Tacrolimus levels were first analyzed as a continuous predictor. Cox models revealed that the risk of graft loss decreased with higher tacrolimus trough levels following dnDSA detection (HR, 0.8, 95% CI, 0.7–0.99; p=0.04; **Table 4**), an association that remained significant following adjustments for age, sex, serum creatinine at detection and time post-transplant at detection (aHR, 0.7; 95% CI 0.6–0.9; p=0.02) as well as following adjustment of antibody removal therapies in addition to these covariates (aHR, 0.7; 95% CI 0.6–0.9; p=0.02). We found was no association between the prednisone dose and graft loss (HR 1.0 95% CI 0.9–1.2, p=0.59), nor between the MMF dose and graft loss (HR 1.0 95% CI 0.999–1.001, p=0.94).

**Table 4:** Univariate and multivariate risk estimates for graft loss associated with tacrolimus levels post dnDSA detection.

	unadjusted		adjusted^a^		adjusted^b^	
	Hazard ratio (95% CI)	p-value	Hazard ratio (95% CI)	p-value	Hazard ratio (95% CI)	p-value
TAC level, continuous (ng/ml)	0.8 (0.7-0.99)	0.04	0.7 (0.6-0.9)	0.02	0.7 (0.6-0.9)	0.02
TAC level^c^						
>6.3 ng/ml	ref	–	ref	–	ref	–
5.3 – 6.3 ng/ml	0.7 (0.2-1.8)	0.41	1.0 (0.3-2.8)	0.96	1.0 (0.3-3.0)	0.99
<5.3 ng/ml	1.9 (0.8-4.2)	0.14	2.9 (1.2-7.5)	0.02	3.0 (1.2-7.5)	0.02

a adjusted for age, sex, serum creatinine at dnDSA detection, time post-transplant at dnDSA detection

b adjusted for age, sex, serum creatinine at dnDSA detection, time post-transplant at dnDSA detection, and antibody removal therapy (plasma exchange and/or IVIG and/or rituximab)

c validation of previously established cut-off (Béland et al. reference 9)

Finally, we aimed to validate the previously established tacrolimus trough cut-off values derived from the tertiles of our previous cohort, which were as follows: <5.3 ng/mL, 5.3–6.3 ng/mL, and >6.3 ng/mL (8). In the adjusted Cox model, individuals with a mean tacrolimus level <5.3 ng/mL had a higher risk of graft loss compared with those with a tacrolimus level >6.3 ng/mL (aHR, 3.0, 95% CI 1.2–7.5; p=0.02). However, there was no significant difference in patients with tacrolimus levels of 5.3–6.3 ng/mL. Taken together, these data validate the association between tacrolimus trough levels and graft loss in this population. It corroborates that tacrolimus levels below 5.3 ng/ml predict poor outcomes in these patients.

### Secondary analyses

We first sought to determine whether restricting the analysis to DSAs with higher MFIs would result in similar observations. We first examined patients using an MFI threshold of 5000 and obtained a limited number of positive DSA for each class: only 1, 16 and 1 patients respectively had DSA of class I only, class II only and both classes using this threshold. We then examined a threshold of 3000. As depicted in **Supplementary Figure S1**, the number of patients with class I only, class II only and both classes with this threshold were 3, 23, and 2 respectively. With the limitation of small numbers in some of these groups, patients with DSA class I did not seem to have a better prognosis than those with class II only.

We next analyzed the impact of antibody removal therapies. First, we focused on the 18 patients with AMR. Among them, 6 patients received plasma exchanges, IVIG and rituximab in accordance to a protocol previously reported by Lefaucheur et al. *(* 20 *)*, 2 patients received the same protocol except for rituximab, and 1 patient received only solumedrol; the remaining 9 patients did not receive additional treatment. **Supplementary Table S2** displays the outcome according to the treatment. The median graft survival for the 10 patients who received solumedrol only or no additional treatment was 43 months post DSA detection, versus 52 months for those who received antibody removal therapy; the difference was not significant (p=0.61). **Supplementary Table S2** shows the proportional hazards model adjusted for age, sex, serum creatinine at DSA detection, and time post-transplant; there was no signal for a difference between groups. Second, we examined the impact of antibody removal therapies according to the HLA class of DSA at the cohort level. We found no impact of such treatment on graft survival for patients with class I only and class II only (**Supplementary Figures S3A, B**); survival was non-significantly better in the four patients with DSA of both classes (**Supplementary Figure S3C**). Given the small number of patients in each subgroup, these results need to be interpreted with caution.

## Discussion

Herein, we examined a cohort of kidney transplant recipients who underwent longitudinal anti-HLA antibody surveillance. The major novel finding was that the risk of graft loss was similar for patients with dnDSAs of HLA class I only and those with dnDSAs of HLA class II only. Second, a tacrolimus level of >5.3 ng/mL was protective in patients with dnDSAs. This effect was robust and remained significant even after adjustment for age, sex, serum creatinine and time post-transplant at detection, and the use of antibody removal therapy.

The observation that dnDSAs of HLA class I may be as detrimental as those of HLA class II contradicts the notion that HLA class II dnDSAs are associated with worse outcomes. However, they are in line with the findings recently reported by Lopez del Moral et al. (21) In a cohort of 400 patients with dnDSAs, they observed a 5-year death-censored allograft survival of 73.4% in patients with HLA class I dnDSAs compared with 79.9% in those with HLA class II dnDSAs. Using a control group comprising patients without dnDSA, we made similar observations and further observed that the risk of graft loss was higher than that in controls. Interestingly, we studied a period similar to that of Lopez del Moral et al., yet observed roughly only one-third of their incidence of dnDSA (400/3344 or 12.0%) (21). Notably, for DSA detection, these authors used an MFI cut-off value of 1000, whereas we used an MFI of 1500 as a guide, combined with eplet analysis. This higher cut-off value may explain why the prevalence of AMR in biopsies performed at the time of dnDSA detection in our center was higher. Herein, 18/34 (53%) of the biopsied patients had AMR (18/55 (33%) of the total population with dnDSA). In contrast, Lopez del Moral et al. observed only 26/400 (6.5%) AMR at the time of dnDSA (21). Given the known association between higher MFI levels, antibody titer, and graft survival (22, 23), it is plausible that using a higher cut-off value enriches the population at risk of histological manifestations and worse clinical outcomes.

Despite a large amount of data being available to guide immunosuppressive therapy at the time of transplant, little information is available in the literature to guide management following the detection of dnDSA. Although anti-HLA antibody screening is recommended, there is currently no strong recommendation about the frequency and duration of surveillance (6). A recent medical decision analysis suggested that the benefit of screening could be offset by a small increase in the risk of death (24). This analysis indicated that the cost effectiveness of screening all patients was marginal at approximately 120 000 to 250 000$ per QALY, but could be more favorable if the monitoring targets patients with low mortality risk but high immunological risk. In contrast, a cohort study that specifically examined rejection rates of a surveillance biopsy program triggered by dnDSA detection observed a 53% rejection rate in patients with stable function (25), a proportion similar to the one observed herein. Another multicenter cohort study indicated a proportion of 41% of subclinical AMR in biopsies performed after dnDSA detection (26). Overall, this suggests a high likelihood of positive biopsy when it is performed in the context of dnDSA surveillance.

A recent prospective, open-labelled, trial with a complex hybrid design tested unblinded biomarker care *versus* standard care in patients with DSA and non-DSA (27). The patients in the intervention group were interviewed to encourage medication adherence and received optimization of tacrolimus. The development of DSA was predictive of graft failure; however, there was no difference between optimized-care and standard care. An important limitation of this study was that the optimization of tacrolimus in the intervention group targeted through levels of 4 to 8 μg/L. This wide range of values included low levels that were shown to be predictive of dnDSA development and of adverse outcomes in patients with dnDSA (8, 12). Therefore, this study did not specifically inform whether or not a higher dose of tacrolimus was beneficial. The results of the current study validated previous observations regarding tacrolimus levels in patients with dnDSAs. Tacrolimus levels <5.3 ng/ml were associated with worse graft survival. Despite several trials confirming that CNI withdrawal or minimization is associated with adverse outcomes (28, 29), tacrolimus levels below 5.5 ng/ml are not uncommon, with an incidence of approximately 20–25% of patients, according to a large longitudinal study (12). In this context, identifying patients with dnDSA to avoid these low levels in high-risk patients may potentially increase the longevity of grafts.

The main strength of this study is the comparison of patients with dnDSA with a control group comprising matched patients without dnDSA. The main limitation, despite the statistically significant results, is that the findings are derived from a relatively small single-center cohort. However, other than the cohort recently studied by Lopez del Moral et al., the size of the cohort analyzed here was comparable to that of previous reports that specifically addressed the outcomes of patients with dnDSA. For instance, Schinstock et al. analyzed outcomes in 54 patients with dnDSA, whereas Wiebe et al. and Parajuili et al. studied 47 and 45 patients respectively (2, 9). In addition, the number of events limited the adjustments for the most important confounding factors. However, using this level of adjustment to control for confounding factors in a cohort with this number of events has been shown to be adequate (30).

In summary, the data presented herein challenge the notion that the prognosis of patients with dnDSAs differs according to HLA class I and HLA class II, suggesting that no distinction should be made when assessing the graft risk in patients with dnDSA. Furthermore, the findings support the notion that low tacrolimus levels in patients with dnDSAs predict graft loss.

## Data Availability

The raw data supporting the conclusions of this article will be made available by the authors, without undue reservation.
