# Supplementary material for: HLA class, calcineurin inhibitor levels, and the risk of graft failure in kidney recipients with de novo donor-specific antibodies
Source: Front Immunol. 2024 Nov 20;15:1493878. doi: 10.3389/fimmu.2024.1493878 (PMC11614807; doi:10.3389/fimmu.2024.1493878)
Supplement: Supplementary file 1 [file DataSheet1.pdf]

Supplemental Figure 1

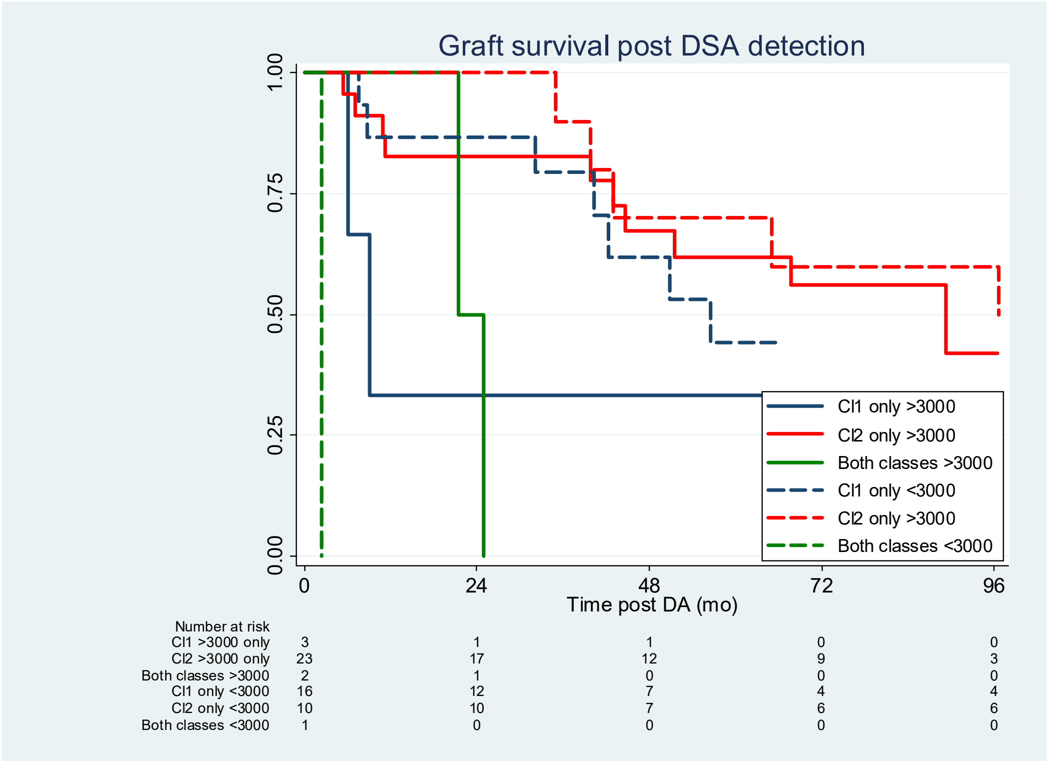

Graft survival for each DSA group, stratified by an MFI threshold of 3000

Supplemental Figure 2

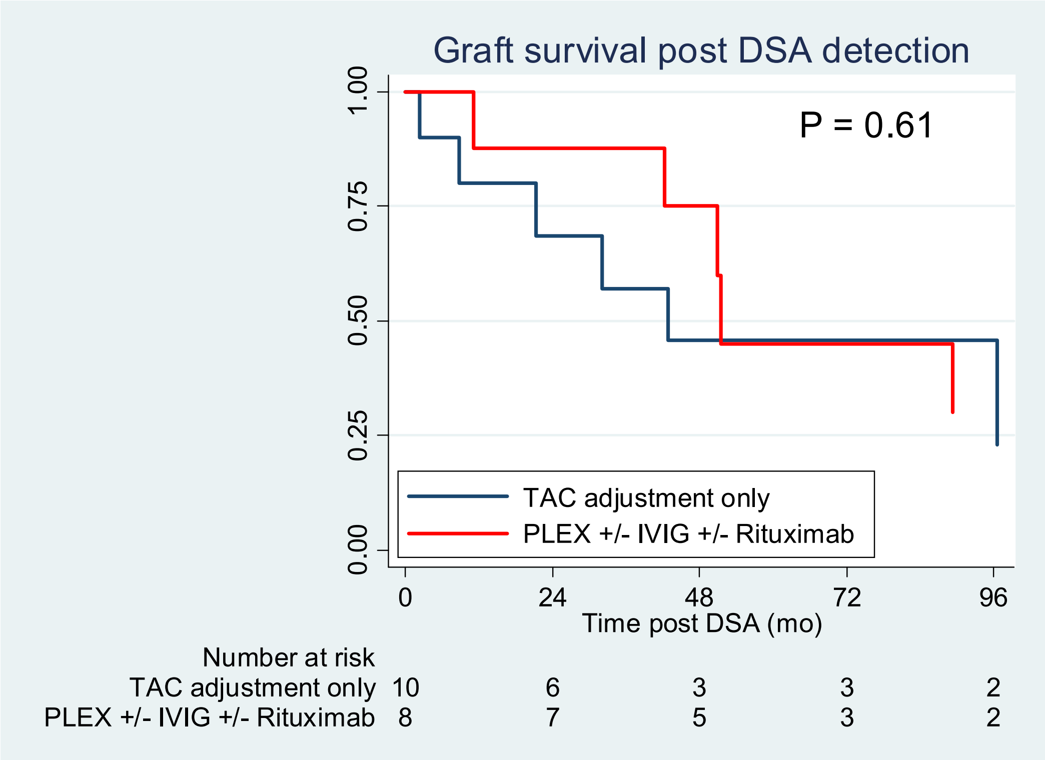

Graft survival for the subset of patients with AMR, by treatment

Supplemental Figure 3

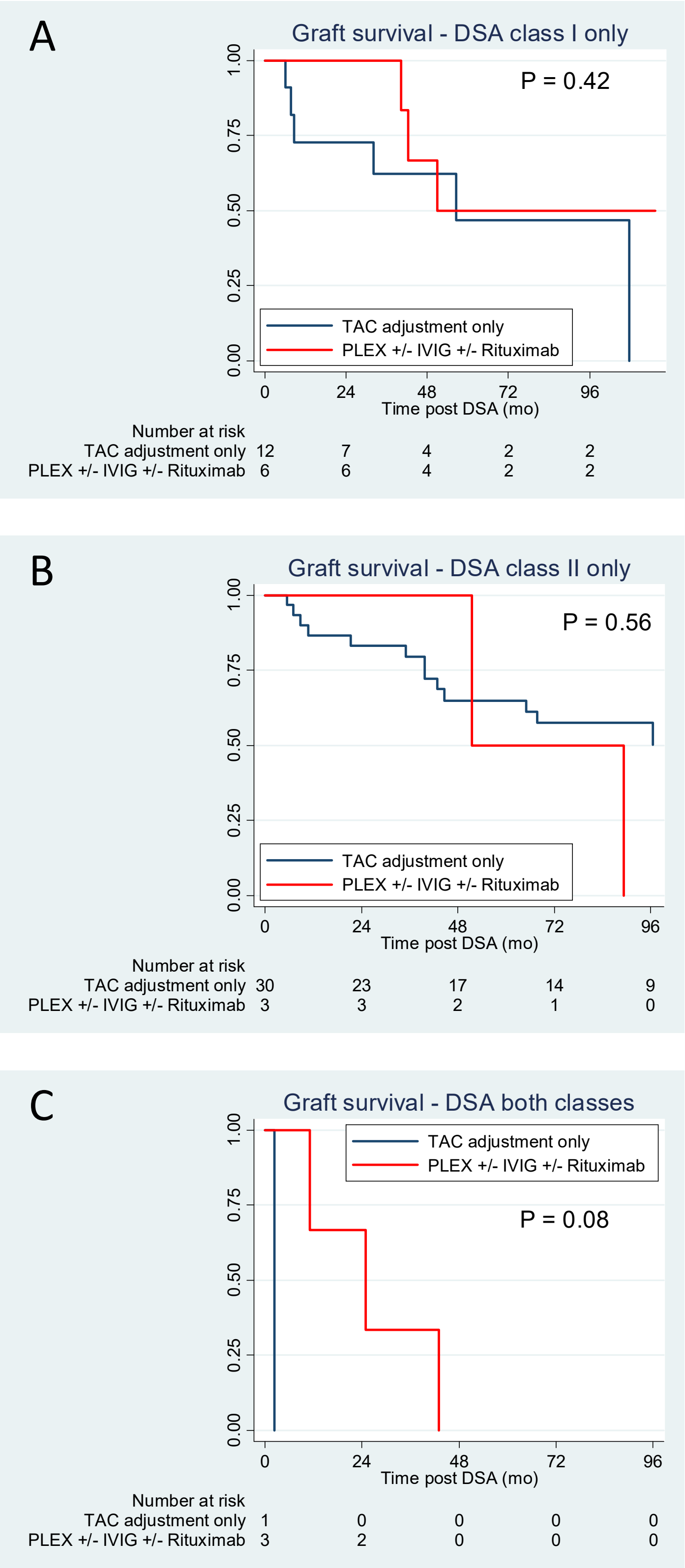

Graft survival by treatment, for each DSA group
