# Supplementary material for: HLA class, calcineurin inhibitor levels, and the risk of graft failure in kidney recipients with de novo donor-specific antibodies
Source: Front Immunol. 2024 Nov 20;15:1493878. doi: 10.3389/fimmu.2024.1493878 (PMC11614807; doi:10.3389/fimmu.2024.1493878)
Supplement: Supplementary file 2 [file DataSheet2.pdf]

SUPPLEMENTAL MATERIAL

Supplemental Table 1: Clinical and pathological characteristics of the dnDSA population and matched controls

	Matched controls[a] (n=51)	Class I only (n=17)	Class II only (n=30)	Classes I and II (n=4)	p-value
Female gender	21 (41)	8 (47)	12 (40)	3 (75)	0.58
Deceased donor	41 (80)	17 (100)	24 (80)	3 (75)	0.25
Serum creatinine (μmol/L)	106 [85, 130]	111 [91, 217]	110 [92, 160]	113 [95, 165]	0.35
HLA mismatch					
A mismatch	1.2 ± 0.8	1.4 ± 0.7	1.1 ± 0.8	1.5 ± 0.6	0.53
B mismatch	1.2 ± 0.7	1.5 ± 0.6	1.1 ± 0.7	1.3 ± 0.5	0.29
DRB1 mismatch	0.8 ± 0.8	0.8 ± 0.8	1.1 ± 0.6	1.0 ± 0.8	0.24
DQB1 mismatch	0.9 ± 0.8	0.5 ± 0.5	1.2 ± 0.8	1.0 ± 0.0	0.15

Data are provided as mean ± SD, n (%) or median [25th, 75th percentiles]. Comparisons were performed using ANOVA or Chi square test.
[a] matched on time of transplant, rank of transplant and recipient age

Supplemental Table 2: Univariate and multivariate risk estimates for graft loss associated with treatment of patients with AMR

	unadjusted		adjusted[a]	
	Hazard ratio (95% CI)	p-value	Hazard ratio (95% CI)	p-value
TAC adjustment only	ref	-	ref	-
PLEX ± IVIG ± Rituximab	0.7 (0.2-2.4)	0.61	1.1 (0.3-4.5)	0.88

[a] adjusted for age, sex, serum creatinine at dnDSA detection, time post transplant at dnDSA detection. n = 18
